# Using 4DCBCT simulation and guidance to evaluate inter-fractional tumor variance during SABR for lung tumor within the lower lobe

**DOI:** 10.1038/s41598-021-99489-1

**Published:** 2021-10-07

**Authors:** Yi Li, Wenjing Wu, Ruixin He, Yongkai Lu, Yuemei Zhang, Long Wang, Xiaozhi Zhang

**Affiliations:** 1 Department of Radiation Oncology, The First Affiliated Hospital of Xi’an Jiaotong University, Xi’an, 710061 China; 2 Department of Radiological Health, Xi’an Center for Disease Control and Prevention, Xi’an, 710054 China

**Keywords:** Radiotherapy, Lung cancer

## Abstract

Inter-fractional tumor variance would lead to insufficient dosage or overdose in tumor region during lung cancer radiotherapy. However, previous works have not considered influence of inter-fractional tumor amplitude variance at treatment position due to lack of effective evaluation method during radiotherapy, especially for lung tumor within the lower lobe. Our objective was to investigate inter-fractional tumor baseline shift and amplitude variance due to respiratory motion with 4DCBCT simulation and guidance during stereotactic ablative body radiotherapy (SABR) for lung tumor. Subject included 19 patients with lung tumor within the lower lobe. 4DCBCT-simulated images at treatment position were acquired sequentially to determine internal tumor volume (ITV) and reference tumor motion at simulation process. Compared with reference tumor motion, 95 4DCBCT-guided images were acquired during each treatment to evaluate inter-fractional tumor baseline shift and amplitude variance, which were − 0.0 ± 1.3 mm and − 0.2 ± 1.4 mm in left–right(LR) direction, 0.9 ± 2.3 mm and 0.4 ± 2.9 mm in superior-inferior (SI) direction, 0.1 ± 1.5 mm and − 0.4 ± 2.0 mm in anterior–posterior (AP) direction. ITV margin were 3.5 mm, 7.5 mm and 5.3 mm in LR, SI and AP directions with van Herk’s (Int J Radiat Oncol Biol Phys 52(5):1407–1422, 2002) formula. 4DCBCT simulation and guidance is a reliable method to evaluate inter-fractional tumor variance during SABR for lung tumor within the lower lobe. ITV margin of 3.5 mm, 7.5 mm and 5.3 mm in LR, SI and AP directions would ensure greater tumor coverage during SABR for lung tumor within the lower lobe.

## Introduction

Stereotactic ablative body radiotherapy (SABR) is an effective treatment procedure allowing the delivery of high radiation doses to lung tumor in a limited number of treatment fractions. The accuracy of each SABR treatment is very important. However, tumor position variance is large due to patient's respiratory movement, which would decrease dose delivery accuracy during lung tumor SABR treatment. Schmidt et al.^1^ demonstrated that patient’s respiratory pattern variance during fractional SABR treatment decreased treatment geometric accuracy and would result in local control decrease and toxicity increase. Accurate respiratory motion management and assessment are crucial during lung tumor SABR treatment, especially for lung tumor within the lower lobe. At present, four-dimensional computed tomography (4DCT) is used to evaluate tumor variance due to respiratory movement at simulation for lung SABR treatment. However, tumor position variance due to respiration motion could vary from fraction to fraction during lung SABR treatment. Rabinowitz et al.^2^ demonstrated the tumor localization deviation caused by respiratory movement was 5.1 mm on average and 5.8 mm on maximum between 4DCT simulation phase and treatment phase for lung tumor. Inter-fractional tumor variance is defined as tumor shift and amplitude variance between every treatment fraction. Previous studies have assessed inter-fractional tumor variance with electric portal image device (EPID)^3^, CBCT guidance and 4DCT imaging^4^. However, these studies mainly focus on inter-fractional tumor baseline shift at the treatment position without evaluating inter-fractional tumor amplitude variance at treatment position due to lack of effective evaluation method during treatment, which maybe result in inaccuracy results during lung SABR radiotherapy.

Recently, 4DCT has been adopt to obtain reference tumor motion at simulation position, four-dimensional cone beam computed tomography (4DCBCT) has been adopt to obtain inter-fractional tumor baseline and amplitude due to patients' respiratory motion during lung tumor radiotherapy^5^, which would make up for the deficiency of EPID, CBCT and 4DCT. However, there are some unsolved issues for evaluating inter-fractional tumor variance with 4DCT simulation and 4DCBCT guidance. Firstly, reference tumor motion obtained from 4DCT scan represents a snapshot in time at simulation^6^. Yang et al.^7^ demonstrated that 4DCT could only collect a limited number of signals of respiratory phase and could not accurately reflect the tumor motion due to respiratory movement. Secondly, previous studies^8,9^ on 4DCBCT-guided radiotherapy have adopted registration between 4DCBCT and average intensity projection (AvgIP) of 4DCT images for tumor variance correction due to respiratory motion, which maybe result in non-homologous image registration and would lead to inaccuracy matched result. Finally, previous studies have not investigated the correlation between inter-fractional baseline shift and inter-fractional amplitude variance. Therefore, the application of 4DCBCT in evaluating inter-fractional tumor variance due to respiratory movement still need to be studied during lung SABR radiotherapy. In this work, in view of above unsolved issues, inter-fractional tumor baseline shifts and amplitude variance are evaluated with 4DCBCT simulation and daily 4DCBCT guidance during every lung SABR treatment. The aim of this study is to provide a method accounting for inter-fractional tumor baseline shift and amplitude variance during lung SABR treatment for non-small cell lung cancer (NSCLC) patients.

## Methods

### Patient characteristics

19 NSCLC SABR patients with peripheral tumor located in the lower lobe were enrolled in the study from May 2018 to January 2020 in the First Affiliated Hospital of Xi'an Jiao Tong University in China. The patient characteristics were listed in Table 1. Volumetric modulated arc therapy (VMAT) technology was adopt to delivery dose to the tumor with prescribed doses of 50 Gy (5 fractions). Neoadjuvant, concurrent or adjuvant chemotherapy was recommended for patients. This study was approved by the Medical Ethical Committee of First Affiliated Hospital of Xi’an Jiaotong University (No: XJTU1AF2020LSK-169). All patients had signed the informed consent form. All methods were conducted in accordance with relevant guidelines and regulations (the ethical standards of the 1964 Declaration of Helsinki and its later amendments or comparable ethical standards).

**Table 1:** Characteristics of the patients (n = 19).

Characteristics	Number
**Sex**	
Male	12
Female	7
**Age (years)**	
Median	48
Range	38–70
Disease stage	I/II NSCLC
**Lung tumor volumes (cm**^**3**^**)**	
Median	301.25
Range	168.95–432.97
**The average BMI (kg/m**^**2**^**)**	
Median	22.2
Range	20.5–23.8
**Side**	
Right	6
Left	13

### 3DCT simulation and preliminary plan

All patients were immobilized using thermoplastic body mold in the supine position with hands raised and arms crossed with elbows on top of the head at simulation. Each patient received a helical three-dimensional computed tomography (3DCT) scanning under free breathing condition on a 16-slices CT scanner (Big bore, Philips Medical Systems, Cleveland, OH). The scanning range was from the thoracic inlet to the whole diaphragm. The scanned images were sent to Monaco V. 5.2y treatment planning system (TPS) (Elekta, Fitchburg, USA) with slice thickness 3 mm and layer spacing 3 mm. Gross tumor volume (GTV) contours were manually delineated by the same professional physician to make the inter-observer lowest as soon as possible with lung window setting (L = − 600Hu, W = 1600Hu). After the physician contoured the GTV on workstation, the physicist determined the treatment center (centroid of GTV) and made preliminary plan with a single vertical field, which was used for the 4DCBCT simulation to determine internal tumor volume (ITV).

### 4DCBCT simulation and ITV determination

All patients were positioned with the same as 3DCT simulation on the Versa HD linear accelerator (Elekta, Crawley, UK) equipped with 4DCBCT, which could obtain patients’ setup and breathing movement information simultaneously. Patients’ position was adjusted from simulation center to treatment center according to preliminary plan setting using laser-based positioning system.4DCBCT images were acquired using X-ray Volume Imaging (XVI) system equipped with the Symmetry Scanned Module. Each 4DCBCT scan took about 1 min to acquire image dates. According to previous study^10^, contract to noise ratio of 4DCBCT image had been shown to increase with slower gantry speed and more projections. In order to decrease image artifacts and motion artifacts on the 4DCBCT images, 975 projections were acquired with scan speed of 3°/s over 200° gantry rotation for image reconstruction, which could compromise between image quality and scan time. The other parameters were as follows: 120 kV, 400mAs, collimator S20, medium resolution, slice thickness 3 mm and layer spacing 3 mm. The acquired projections were binned retrospectively into 10 phased-angle sorting images though tracking the motion of patients’ diaphragm. The average intensity projection (AvgIP) image could be created through 10 phase-sorted 4DCBCT datasets. The 4DCBCT images were registered with the reference 3DCT image. Two separate dual registration was done. Firstly, a rectangular clipbox for automatic bone registration between reference 3DCT and AvgIP image from 4DCBCT-guided images in the XVI software was confined around the vertebrae and bone structure of affected lung side for setup correction. Secondly, a mask was generated for automatic image registration of GTV: only the volume of reference 3DCT within this mask was used in the XVI software for automatic soft-tissue registration. The mask was generated by expansion of the GTV with 5 mm margin and manual exclusion of all bony structures (ribs, sternum, and vertebrae) using a drawing tool. Automatic soft-tissue registration between the volume of 3DCT inside the mask and all 10 phases of 4DCBCT was performed: the position of the tumor was identified in each breathing phase. After setup error was corrected completely by first registration, 10 phase-sorted 4DCBCT images were transmitted to TPS for 10 phase GTVs contouring. The ITV was generated by performing the union of 10 GTVs contour based on 4DCBCT images. ITV was copied to the 3DCT for planning. An isotropic margin of 5 mm was added to ITV to form planning target volume (PTV), which would account into treatment inaccuracies^11^.

### Treatment planning

The treatment planning was done on the 3DCT. All patients were prescribed with a hypo-fractionated treatment regime (50 Gy in 5 fractions), which delivered dose with prescribed dose covering the 99% ITV volume and 95% PTV volume. In order to reduce the impact of long treatment time on the accuracy of treatment, 6FFF high dose rate (1400 cGy/min) energy and partial double-arc VMAT technology were adopt to delivery prescribed dose with the gantry angle range of 180° ± 20°, which resulted in treatment time about 3 min^12^. Treatment center was set as same as 3DCT simulation. During plan parameter optimization, the contralateral lung bock was created to fully shied radiation, which would reduce low dose in this area by limiting the maximum dose in the optimization condition. After treatment plan was completed, reference AvgIP image from 4DCBCT-simulated images and all structures had been transmitted to the XVI system for image registration during every lung SABR treatment.

### Pre-treatment 4DCBCT scanning

**Figure 1:**
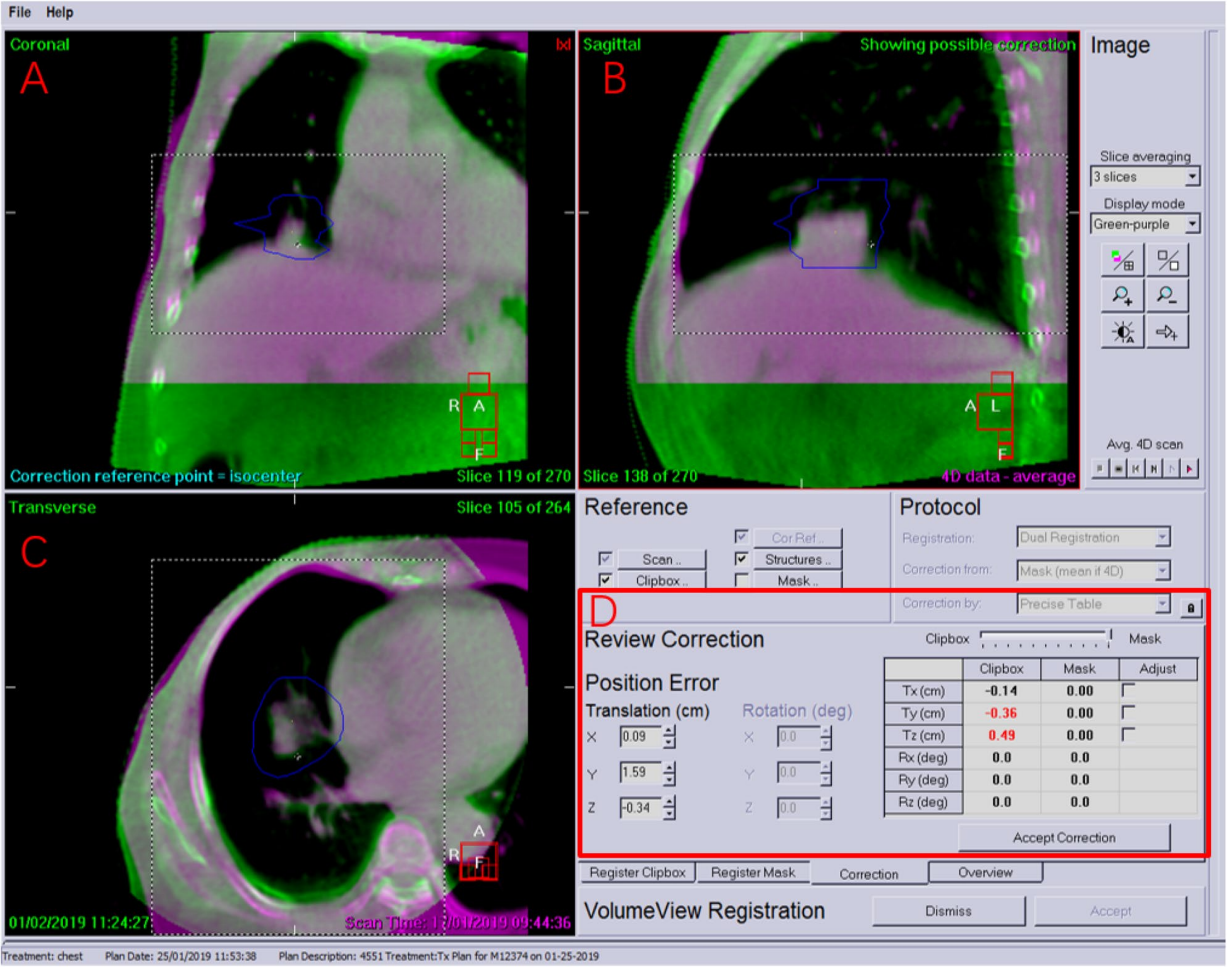
Registration result between reference AvgIP (green color) from 4DCBCT-simulated images and AvgIP (purple color) from 4DCBCT-guided images (**A**: Coronal plane, **B**: Sagittal plane, **C**: Transverse plane, **D**: Clipbox and Mask registered results).

**Figure 2:**
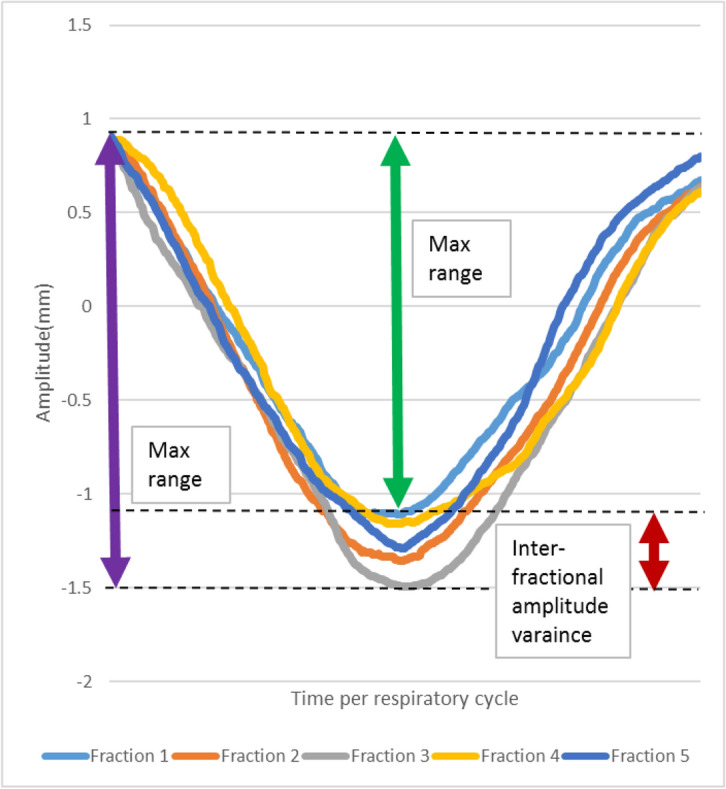
Measure of the inter-fractional tumor amplitude variance in SI direction for five fractions treatment.

All patients were treated with VersaHD accelerator (Elekta Medical Systems) equipped with 4DCBCT. The patients were instructed to lie down on the same posture as that during 4DCBCT simulation, and the position was aligned with treatment center using laser-based positioning system. 4DCBCT imaging was performed with parameter as same as 4DCBCT simulation before lung SABR treatment. The 4DCBCT image was registered with reference AvgIP image from 4DCBCT-simulated images with the dual registration (Fig. 1). Two separate dual registration was done. Firstly, a rectangular clipbox for automatic bone registration between reference AvgIP and AvgIP from 4DCBCT-guided images in the XVI software was confined around the vertebrae and bone structure of affected lung side for setup correction. Secondly, a mask was generated for automatic image registration of ITV: only the volume of reference AvgIP within this mask was used in the XVI software for automatic soft-tissue registration. The mask was generated by expansion of the ITV with 5 mm margin and manual exclusion of all bony structures (ribs, sternum, and vertebrae) using a drawing tool. Automatic soft-tissue registration between the volume of reference AvgIP inside the mask and all 10 phases of 4DCBCT guidance was performed for breath motion correction. The dual registration results were reviewed by the same professional physician and manually adjusted if PTV could not cover total current tumor. Registration results were used to shift the table along the three directions. The baseline was defined as average tumor position in all directions. The baseline shift was measured by subtracting the clipbox suggested setup correction from the applied table shift. The inter-fractional baseline shift variance was calculated by subtracting following baseline shift from baseline shift at first fraction treatment. Tumor amplitude for each fraction was quantified as peak-to-peak motion range during 4DBCT scanning, which was calculated by XVI software. The inter-fractional amplitude variance was evaluated by calculating the amplitude variance range among all treatment fractions (Fig. 2) (maximum amplitude range at first fraction as benchmark) and then extracting the corresponding amplitude variance in left–right (LR) direction, superior-inferior (SI) direction and anterior–posterior (AP) directions for all treatment fractions.

### Statistical analysis

All statistical analysis was performed by SPSS Statistics V22.0 software (IBM Corp., Armonk, NY). Quantitative data was expressed as mean ± standard deviation (SD). Paired T test was used for correlation test between tumor variance in three directions. Pearson correlation test was used for correlation test between inter-fractional baseline shift variance and amplitude variance. Differences were considered significant for P < 0.05. The population systematic error component (©) was calculated as the standard deviation of mean error of inter-fractional tumor variance for each patient, whereas the root mean square of the standard deviation represented the population random error component ()^13,14^. The corresponding ITV margin was calculated with formulas reported by ICRU 83^15^.

## Results

### Inter-fractional ITV baseline shift variance

**Figure 3:**
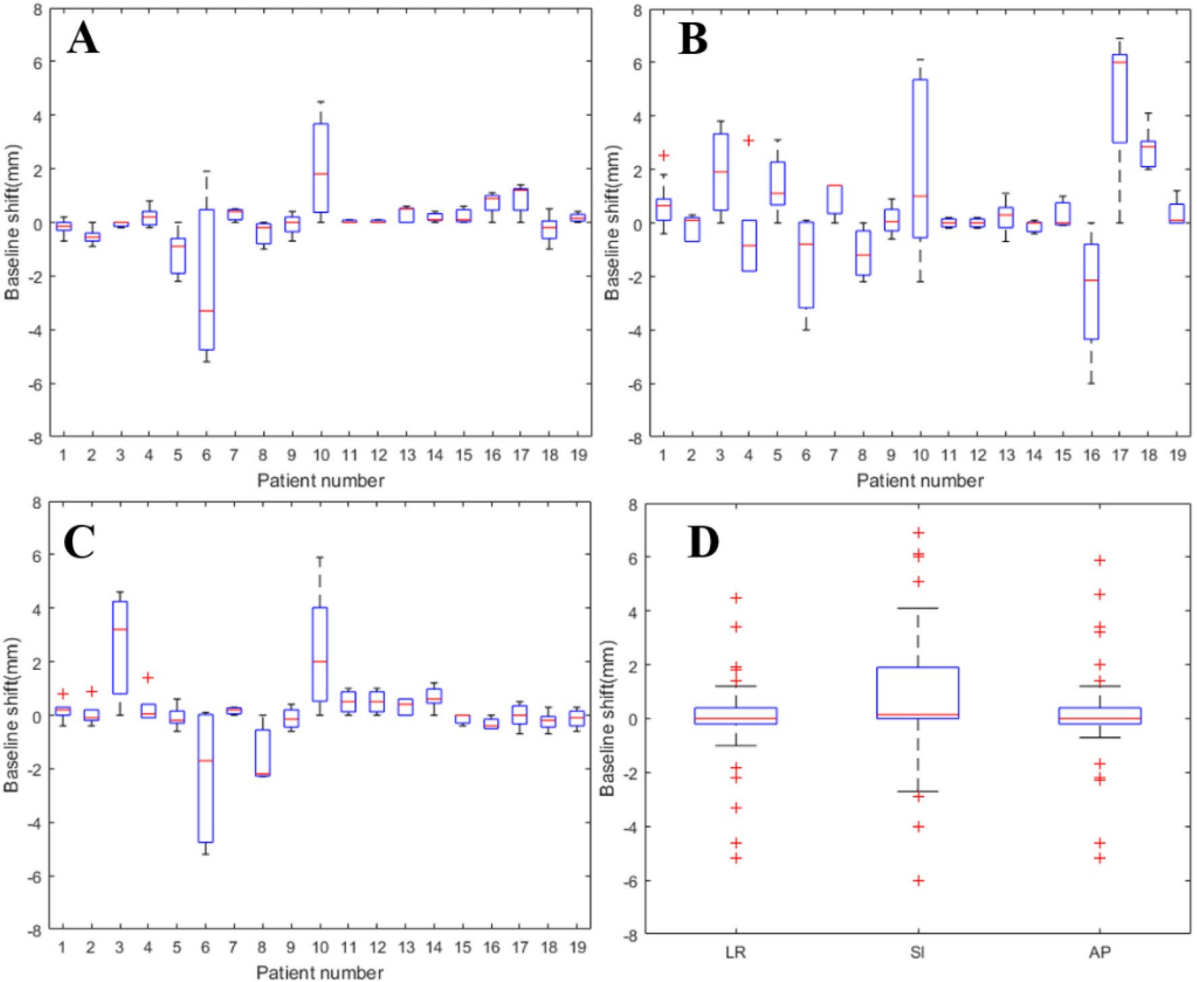
Inter-fractional tumor baseline shift in LR, SI and AP directions for 19 patients (**A**: values in LR direction, **B**: values in SI direction, **C**: values in AP direction, **D**: statistic values in three directions).

The distribution of inter-fractional ITV baseline shift variance for all patients was shown in Fig. 3. Inter-fractional ITV baseline shift variances were − 0.0 ± 1.3 mm (range: − 5.2 to 4.5 mm) in LR direction, 0.9 ± 2.3 mm (range: − 6.0 to 6.9 mm) in SI direction and 0.1 ± 1.5 mm (range: − 5.2 to 5.9 mm) in AP direction, which all followed normal distribution. The inter-fractional baseline shift variance had a greater value in SI direction than that in other directions. (SI vs AP: t = 3.485, P = 0.001; SI vs RL: t = 3.003, P = 0.004). © and  were 0.6 mm and 0.7 mm, 0.5 mm and 0.6 mm, 1.0 mm and 0.4 mm in LR, SI and AP directions respectively. The frequency of inter-fractional baseline shift variance range ≥ 2 mm was 6.38%, 30.85% and 9.57% in LR, SI and AP directions respectively. The frequency of inter-fractional baseline shift range ≥ 5 mm was 6.38% in SI direction. The largest inter-fractional baseline shift variance was 6.9 mm along superior direction observed for patient 17 in fourth treatment fraction.

### Inter-fractional ITV amplitude variance

**Figure 4:**
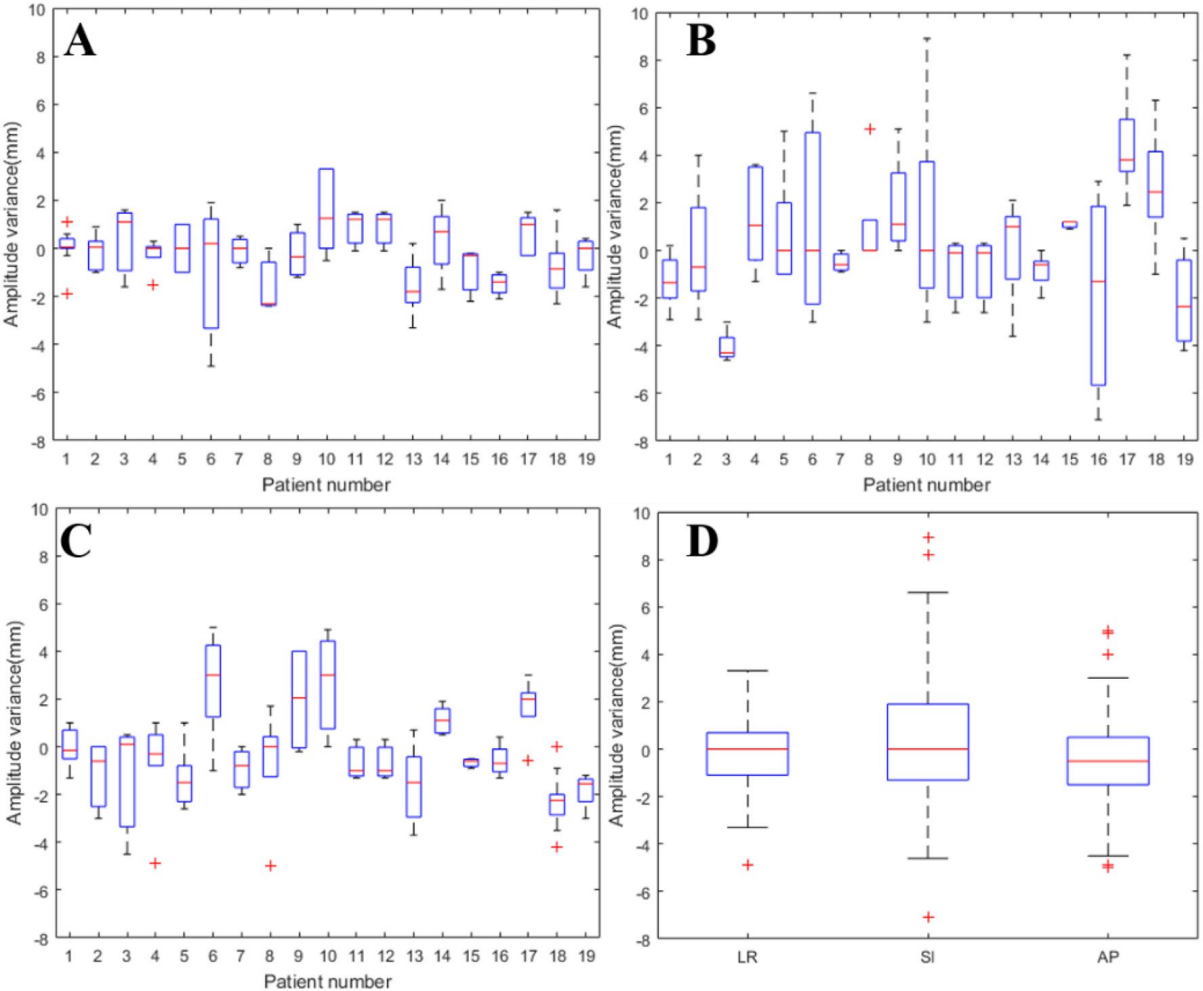
Inter-fractional tumor amplitude variance in LR, AP, and SI directions for 19 patients (**A**: values in LR direction, **B**: values in SI direction, **C**: values in AP direction, **D**: statistic values in three directions).

The distribution of inter-fractional ITV amplitude variance for all patients was shown in Fig. 4. Inter-fractional amplitude variance across all fractions were − 0.2 ± 1.4 mm (range: − 5.0 to 3.3 mm) in LR direction, 0.4 ± 2.9 mm (range: − 7.1 to 8.9 mm) in SI direction and − 0.4 ± 2.0 mm (range: − 5.0 to 5.0 mm) in AP direction, which all followed normal distribution. ITV amplitude variance had a greater value in SI direction than that in other directions (SI vs LR: t = 2.127, P = 0.036; SI vs AP: t = 2.330, P = 0.022). © and  were 0.9 mm and 0.9 mm, 1.2 mm and 1.2 mm, 1.6 mm and 0.8 mm in LR, SI and AP directions respectively. The frequency of inter-fractional amplitude variance range ≥ 2 mm was 12.77%, 42.55% and 32.98% in LR, SI and AP direction respectively. The frequency of inter-fractional amplitude variance range ≥ 5 mm was 9.57% in SI direction. The largest inter-fractional amplitude variance was 8.9 mm in superior direction (patient 10) due to patient’s sudden deep inspiration in first treatment fraction.

### Correlation between inter-fractional baseline shift variance and amplitude variance

Inter-fractional baseline shift variance was positively correlated with inter-fractional amplitude variance in SI direction (r = 0.353 P = 0.000). Inter-fractional baseline shift variance was negatively correlated with inter-fractional amplitude variance in AP direction (r = − 0.227 P = 0.027). However, inter-fractional baseline shift variance had no correlated with inter-fractional amplitude variance in LR direction (r = − 0.017 P = 0.872).

### ITV margin

To compensate inter-fractional tumor baseline shift and amplitude variance, maximum ITV margin were 3.5, 7.5 and 5.3 mm in LR, SI and AP directions calculated with formula by VanHerk et al.^16^ (Table 2).

**Table 2:** ITV margin in three directions.

Author	Formula	ITV margin (mm)			The statistical assumption
		LR	SI	AP	
Stroom^13^	$2\sum { + 0.7\sigma }$	3.0	6.3	4.5	95% absorbed dose to on average 99% of CTV tested in realistic plans
vanHerk^14^	$2.5\sum { + 0.7\sigma }$	3.5	7.5	5.3	Minimum absorbed dose to CTV is 95% for 90% of patients. Analytical solution for perfect conformation
Parker^17^	$\sum { + \sqrt {\left( {\sigma^{2} + \sum {^{2} } } \right)} }$	2.7	5.7	4.0	95% minimum absorbed dose and 100% absorbed dose for 95% of volume. Probability levels not specified
vanHerk^18^	$\sqrt {2.7^{2} \sum {^{2} + 1.6^{2} \sigma^{2} } } - 0.28\;{\text{cm}}$	0.7	4.6	2.4	Monte Carlo based test of 1% TCP loss due to geometrical errors

## Discussion

It has been confirmed that patient’s respiratory motion has a significant impact on the dose distribution of tumor area and surrounding normal tissues during lung SABR treatment^19^. 4DCBCT imaging could obtain 10 phased-angle sorting images based on a respiratory signal, which is extracted from the diaphragm position motion and proved to be a robust method for binning^20,21^. Moreover, 4DCBCT simulation and guidance could collected about 80 patients’ respiratory motion cycles for each scanning, which provides accuracy tumor motion information for homologous image registration between 4DCBCT-simulated image and 4DCBCT-guided image during lung SABR treatment. This study has evaluated inter-fractional tumor baseline shifts and amplitude variance due to respiratory motion with 4DCBCT simulation and guidance during lung SABR treatment for tumor within the lower lobe.

The larger inter-fractional baseline shift variance along the SI direction observed in this study was consistent with work by Pini et al.^8^. They demonstrated absolute value range of inter-fractional baseline shift were 0–5.6 mm and 0–5.7 mm in LR and AP directions using 4DCT simulation and 4DCBCT guidance during lung stereotactic body radiation therapy (SBRT) treatment, which was similar with results in this study (0–5.2 mm and 0–5.9 mm in LR and AP directions). However, the absolute value range in SI direction (0–7.8 mm) with work by Pini S was greater than the result (0–5.9 mm) in this study. It was maybe caused by non-homologous image registration between 4DCT image and 4DCBCT image, which could not accurately reflect the tumor baseline variance during fractional treatment and resulted in greater inter-fractional baseline shift along the SI direction than the results in this study. The larger inter-fractional amplitude shift was along the SI direction observed in this study, which was consistent with data from other reporting result using 4DCT simulation and daily pre-treatment 4DCT guidance at simulation position during lung SBRT^22^, which demonstrated that homologous image registration maybe accurately reflect inter-fractional tumor amplitude variance. Pini et al.^9^ demonstrated the mean value of inter-fractional amplitude variance was 2.8 mm, which was greater than results (0.44 mm) in this study. It was maybe due to a limited number of signals of respiratory phase collected by 4DCT simulation in work by Pini et al., which could not completely reflect the tumor movement amplitude of the patient during fractional treatment^7^ and resulted in larger inter-fractional amplitude variance along the SI direction with 4DCT simulation and 4DCBCT guidance.

In the inter-fractional baseline shift variance along SI direction, three patients (P10, P16, and P17) had greater values with more than 20% of treatment fractions with shifts above 5 mm. It was mainly related to large breath amplitude. These patients were young obese patients with great respiratory movement and tumor adhered to diaphragm in these patients, which resulted in tumor variance greatly affected by the deep or shallow respiration and ultimately greater inter-fractional baseline shift variance. Moreover, in the inter-fractional baseline shift variance along LR or AP direction, two patients (P6, P10) had greater values, which might be due to changes in breathing patterns and ultimately resulted in greater variance in LR or AP direction. Similar results were found in inter-fractional amplitude variance of four patients (P6, P10, P16, and P17) with more than 40% of treatment fractions with shifts above 5 mm along SI direction, which was also mainly related to large breath amplitude. In addition, in the inter-fractional amplitude variance along LR or AP direction, same patients (P6, P10) had greater values.

Inter-fractional tumor variance due to respiratory motion also depended on different fixed device and anatomic tumor location. Ueda et al.^3^ demonstrated ITV margin was 6.4 mm along SI direction based on inter-fractional tumor variance with BodyFix double-vacuum immobilization system, which was less than ITV margin in our result (7.5 mm) along SI direction with thermoplastic body mold immobilization device. It was maybe due to better fixation with BodyFix double-vacuum immobilization system^23,24^, which resulted in less ITV margin. Moreover, work by Ueda et al.^3^ demonstrated inter-fractional tumor motion variance due to respiratory motion could only be evaluated along SI direction with an EPID in cine mode and might underestimate inter-fractional tumor motion variations. Sun et al.^4^ had evaluated inter-fractional baseline shift variance immobilized with evacuated bags and demonstrated the greater variance was along the AP direction. It was maybe caused by less fixation along AP direction with evacuated bags immobilization device for patients’ BMI < 24^25^, which resulted in greater inter-fractional baseline shift along AP direction. Moreover, Sun et al.^4^ demonstrated that subjective bias could be induced by non-homologous image registration between 4DCT image and CBCT image, which might influence the inter-fractional baseline shift and final ITV margin result. Atkins^22^. demonstrated tumors in the lower lobe had increased inter-fractional tumor motion in the SI direction compared to tumor in the upper or middle lobe.

Evidence suggested that motion management were essential for the safe delivery of SBRT^26^. There were many motion management techniques including respiratory gating, active breathing control (ABC), deep inspiration breath hold (DIBH), abdomen compression and so on. Yoshihiro et al.^27^ demonstrated the inter-fractional tumor motion variance were 2.1 ± 2.9 mm and 2.1 ± 2.2 mm during DC50 (gating window from 30 to 70%) and DC30 (gating window from 40 to 60%) in SI direction with respiratory gating technique for lung SBRT patients. However, three issues limited widespread adoption for respirator gating^26^. First, respiratory gating prolonged delivery time that could affect its clinical tolerability. Secondly, there was the potential residual tumor motion within the temporal gating. Finally, respiratory gating could rely heavily on consistency of respiratory motion over time. However, the relationship between tumor position and chest wall/fiducial position might drift, resulting in systematic uncertainty in the trigger signal and potentially increasing the risk of geometric miss. Zhong et al.^28^ demonstrated the use of ABC decreased tumor motion amplitude to less than 1 mm in three directions. However, systematic and random errors of inter-fractional tumor baseline shift were 2.3/1.9 mm, 3.4/4.1 mm and 2.5/2.8 mm in LR, SI and AP directions respectively, which was larger than our results. Larger inter-fractional tumor baseline shift with ABC was might affected by two causes: (1) the delay between the activation of ABC when the threshold volume was reached and the closure of balloon valve caused a variation in the lung volume^29^; (2) difference in the residual volume prior to inspiration when performing the breath-hold. Barrett et al.^30^ demonstrated inter-fractional tumor position with DIBH were 0.7 ± 1.2 mm, 3.1 ± 2.6 mm and 1.6 ± 1.8 mm in LR, SI and AP directions respectively for lung SABR treatment, which was less than our results. Zhang et al.^31^ demonstrated the use of abdominal compression decreased inter-fractional tumor position variation in SI direction, but it seemed to increase inter-fractional tumor position variation in LR and AP directions for lung SBRT treatment. Although ABC, DIBH and abdominal compression could reduce tumor motion amplitude during lung SBRT treatment in all three directions, this restrained and forced breath type also be difficult to tolerate for all patients or for the entire course of radiotherapy. Despite the variety of approaches implemented for motion management, none of above mentioned techniques were a one-size fits all solution.

Although the ITV margin depended on imaging method, fixed device and motion management, many other studies on SBRT had adopted 5 mm as the ITV margin according for inter-fractional tumor variance due to respiratory motion^32–34^. However, in these reports, specific reason for using 5 mm was never stated and an appropriate margin was unclear. In this study, based on inter-fractional baseline shift and amplitude variance using 4DCBCT simulation and daily 4DCBCT guidance, maximum ITV margin was calculated as 3.5, 7.5 and 5.3 mm in LR, SI and AP directions respectively with formula by vanHerk^16^, which indicated that an isotropic margin of 5.0 mm might not be sufficient along SI and AP directions. Many authors had proposed formulas to calculate the target margin according to different statistical assumption as shown in Table 2. Among all margin formulas in Table 2, the statistical assumption with van Herk’s^14^ formula was that minimum absorbed dose to CTV was 95% for 90% of patients, which met our clinical requirements. The requirement about minimum absorbed dose was high demand in clinical treatment plans, so the bigger margin with van Herk’s^14^ formula was needed to meet the requirement. It was consistent with the result in my study, which showed that among all formulas, the maximum margin was calculated with the formula by van Herk^14^. The statistical assumption with van Herk’s^18^ formula was that Monte Carlo based test of 1% TCP loss due to geometrical errors. The results in my study showed that the margin calculated with van Herk’s^18^ formula was less than one with other formulas. However, a relevant result emerging from our analysis was that ITV margin accounting for inter-fractional tumor variance due to respiratory motion was highly patient-specific and anisotropic.

## Conclusion

4DCBCT imaging is appropriate to account for the inter-fractional tumor baseline shift and amplitude variation due to respiration motion and allows accurate evaluation of ITV margin during lung SABR treatment. The inter-fractional tumor motion variance observed in this study suggests an ITV margin of 3.5, 7.5 and 5.3 mm in LR, SI and AP directions would ensure greater tumor coverage during lung SABR treatment. Moreover, the data in this study is only from one department, which means that the ITV margin in this study could not apply to data with different situations. A larger dataset and more data sources could make the result more robust and better generalization capability.

## References

[1] Schmidt ML, Hoffmann L, Kandi M, Møller DS, Poulsen PR (2013). Dosimetric impact of respiratory motion, interfraction baseline shifts, and anatomical changes in radiotherapy of non-small cell lung cancer. Acta Oncol..

[2] Rabinowitz I, Broomberg J, Goitein M, McCarthy K, Leong J (1985). Accuracy of radiation field alignment in clinical practice. Int. J. Radiat. Oncol. Biol. Phys..

[3] Ueda Y, Miyazaki M, Nishiyama K, Suzuki O, Tsujii K, Miyagi K (2012). Craniocaudal safety margin calculation based on interfractional changes in tumor motion in lung SBRT assessed with an EPID in cine mode. Int. J. Radiat. Oncol. Biol. Phys..

[4] Sun Y, Ge H, Cheng S (2016). Evaluation of interfractional variation of the centroid position and volume of internal target volume during stereotactic body radiotherapy of lung cancer using cone-beam computed tomography. J. Appl. Clin. Med. Phys..

[5] Bellec J, Arab-Ceschia F, Castelli J, Lafond C, Chajon E (2020). ITV versus mid-ventilation for treatment planning in lung SBRT: A comparison of target coverage and PTV adequacy by using in-treatment 4D cone beam CT. Radiat. Oncol..

[6] Steiner E, Shieh CC, Caillet V (2018). 4-Dimensional cone beam computed tomography-measured target motion underrepresents actual motion. Int. J. Radiat. Oncol. Biol. Phys..

[7] Yang M, Timmerman R (2018). Stereotactic ablative radiotherapy uncertainties: Delineation, setup and motion. Semin. Radiat. Oncol..

[8] Pini S, Russo S, Esposito M, Paoletti L, Bastiani P (2018). 130. Inter-fraction variability of respiratory-induced tumour motion in lung SBRT: 4D-CBCT. Physica Med..

[9] Pini S, Gala GD, Russo S, Esposito M, Bastiani P (2019). EP-1995 Anisotropic definition of ITV-PTV margins according to the target position in lung SBRT with 4DCBCT. Radiother. Oncol..

[10] Santoso AP, Song KH, Qin Y (2016). Evaluation of gantry speed on image quality and imaging dose for 4D cone-beam CT acquisition. Radiat. Oncol..

[11] Reitz D, Carl G, Schönecker S (2018). Real-time intra-fraction motion management in breast cancer radiotherapy: Analysis of 2028 treatment sessions. Radiat. Oncol..

[12] Wiant DB, Wentworth S, Maurer JM (2014). Surface imaging-based analysis of intrafraction motion for breast radiotherapy patients. J. Appl. Clin. Med. Phys..

[13] Stroom JC, de Boer HC, Huizenga H (1999). Inclusion of geometrical uncertainties in radiotherapy treatment planning by means of coverage probability. Int. J. Radiat. Oncol. Biol. Phys..

[14] van Herk M, Remeijer P, Rasch C (2000). The probability of correct target dosage: Dose-population histograms for deriving treatment margins in radiotherapy. Int. J. Radiat. Oncol. Biol. Phys..

[15] Hodapp N (2012). The ICRU Report 83: Prescribing, recording and reporting photon-beam intensity-modulated radiation therapy (IMRT). Strahlenther. Onkol..

[16] van Herk M (2004). Errors and margins in radiotherapy. Semin. Radiat. Oncol..

[17] Parker BC, Shiu AS, Maor MH (2002). PTV margin determination in conformal SRT of intracranial lesions. J. Appl. Clin. Med. Phys..

[18] van Herk M, Remeijer P, Lebesque JV (2002). Inclusion of geometric uncertainties in treatment plan evaluation. Int. J. Radiat. Oncol. Biol. Phys..

[19] Court LE, Seco J, Lu XQ (2010). Use of a realistic breathing lung phantom to evaluate dose delivery errors. Med. Phys..

[20] Sonke JJ, Zijp L, Remeijer P (2005). Respiratory correlated cone beam CT. Med. Phys..

[21] Purdie TG, Moseley DJ, Bissonnette JP (2006). Respiration correlated cone-beam computed tomography and 4DCT for evaluating target motion in Stereotactic Lung Radiation Therapy. Acta Oncol..

[22] Atkins KM, Chen Y, Elliott DA (2015). The impact of anatomic tumor location on inter-fraction tumor motion during lung stereotactic body radiation therapy (SBRT). J. Radiosurg. SBRT..

[23] Hubie C, Shaw M, Bydder S (2017). A randomised comparison of three different immobilisation devices for thoracic and abdominal cancers. J. Med. Radiat. Sci..

[24] Han K, Cheung P, Basran PS, Poon I, Yeung L, Lochray F (2010). A comparison of two immobilization systems for stereotactic body radiation therapy of lung tumors. Radiother. Oncol..

[25] Chen G, Dong B, Shan G (2019). Choice of immobilization of stereotactic body radiotherapy in lung tumor patient by BMI. BMC Cancer.

[26] Molitoris JK, Diwanji T, Snider JW (2018). Advances in the use of motion management and image guidance in radiation therapy treatment for lung cancer. J. Thorac. Dis..

[27] Ueda Y, Oohira S, Isono M, Miyazaki M, Teshima T (2016). Asymmetric margin setting at the cranial and caudal sides in respiratory gated and non-gated stereotactic body radiotherapy for lung cancer. Br. J. Radiol..

[28] Zhong R, Wang J, Lin Z, Feng X, Lu Y (2014). Implementation of single-breath-hold cone beam CT guided hypofraction radiotherapy for lung cancer. Radiat. Oncol..

[29] Mcnair HA, Brock J, Symonds-Tayler J (2009). Feasibility of the use of the Active Breathing Co ordinator (ABC) in patients receiving radical radiotherapy for non-small cell lung cancer (NSCLC). Radiother. Oncol..

[30] Barrett S, Taylor A, Rock L (2017). Evaluation of a reproducible breath hold technique for the SABR treatment of lower lobe lung tumours. J. Radiother. Pract..

[31] Zhang, M., Jiang, R. & Zhan, L. Cone-Beam CT assessment of inter-fraction and intra-fraction motions during lung stereotactic body radiotherapy with and without abdominal compression. In *World Congress on Medical Physics and Biomedical Engineering, June 7-12, 2015, Toronto, Canada* (2015.).

[32] Hara R, Itami J, Kondo T (2006). Clinical outcomes of single-fraction stereotactic radiation therapy of lung tumors. Cancer.

[33] Koto M, Takai Y, Ogawa Y (2007). A phase II study on stereotactic body radiotherapy for stage I non-small cell lung cancer. Radiother. Oncol..

[34] Yeung AR, Li JG, Shi W (2009). Tumor localization using cone-beam CT reduces setup margins in conventionally fractionated radiotherapy for lung tumors. Int. J. Radiat. Oncol. Biol. Phys..

